# A Treat-to-Target Update in Ulcerative Colitis: A Systematic Review

**DOI:** 10.14309/ajg.0000000000000183

**Published:** 2019-06-06

**Authors:** Ryan Ungaro, Jean-Frédéric Colombel, Trevor Lissoos, Laurent Peyrin-Biroulet

**Affiliations:** 1 Icahn School of Medicine at Mount Sinai, New York, New York, USA;; 2 Takeda Pharmaceuticals USA, Inc., Deerfield, Illinois, USA;; 3 Department of Hepato-Gastroenterology and Inserm U954, University Hospital of Nancy, University of Lorraine, Vandoeuvre-lès-Nancy, France.

## Abstract

**OBJECTIVES:**

In 2015, the Selecting Therapeutic Targets in Inflammatory Bowel Disease (STRIDE) program proposed shifting the therapeutic focus on ulcerative colitis (UC) toward altering the natural history of the disease course by regularly monitoring objective measurements of disease activity and tailoring treatment accordingly. The therapeutic paradigm shift was well received in the research community and is often cited. However, new evidence on optimal UC treatment targets continues to accumulate since the time of the STRIDE guidelines. This systematic review summarizes the evidence accrued since the STRIDE UC recommendations, discusses the barriers for adoption of treat-to-target approaches in clinical practice in UC, and suggests directions for future research.

**METHODS:**

We systematically reviewed MEDLINE for studies from the time of the STRIDE systematic review up to March 31, 2018, that assessed the potential treatment targets identified by the STRIDE recommendations.

**RESULTS:**

Each potential treatment target literature search returned > 200 articles, which were then reviewed by 2 independent investigators for relevant studies. Selected studies of clinical factors, patient-reported outcomes, endoscopy, histology, imaging, and biomarkers and implications on treatment targets are summarized.

**CONCLUSIONS:**

It appears that the relative weight given to different therapeutic targets in the development and improvement of UC treatments could be optimized, with an increased emphasis on endoscopic and histological targets over clinical or symptomatic targets. For this evolution to occur, however, new research has to demonstrate that the treat-to-target approach will deliver on the promise of better long-term outcomes compared with current approaches.

## INTRODUCTION

Ulcerative colitis (UC) is a chronic disease with a remitting and relapsing course that can progress from asymptomatic mild inflammation to extensive inflammation of the colon, resulting in frequent bloody stools, colonic motility dysfunction, potentially permanent fibrosis and tissue damage, systemic symptoms, and the need for surgery (1–5). Approximately one-third (31%) of patients with limited UC at diagnosis will have disease extension by 10 years (6). In 10%–15% of patients, UC can ultimately lead to colectomy (7). Achieving mucosal healing *via* treatment lowers the risk of requiring colectomy (7–9), which is important, because colectomy provides symptomatic relief, but no cure, and is associated with complications in up to a third of patients (10). UC management focused on symptomatic control may leave less active or smoldering disease (i.e., mucosal healing unachieved) lingering, risking future relapse (11). Despite an improving treatment landscape, long-term colectomy rates have not declined over a 10-year period (7), highlighting the need for new therapeutic strategies.

In 2015, the Selecting Therapeutic Targets in Inflammatory Bowel Disease (STRIDE) committee defined the treat-to-target (T2T) approach for inflammatory bowel disease (IBD), which shifted the goal of UC treatment to long-term prevention of disease complications (dysplasia/cancer, hospitalizations, colectomy) and proposed monitoring of objective disease activity measurements (e.g., endoscopic evidence of inflammation) (2,12,13). The T2T approach, adapted from the rheumatoid arthritis paradigm, aims to achieve disease remission by adjusting therapy according to the achievement (or not) of predefined treatment response targets (2,9,13). The STRIDE committee proposed a composite target of normalization of bowel habits and intestinal inflammation, but evidence was limited for the incorporation of histology and biomarker targets (13).

Recent evidence indicates that complete mucosal healing might be the ideal therapeutic goal in UC (14). As the definition of mucosal healing continues to evolve, 2 aspects should be considered: endoscopic and histological healing because evidence suggests that microscopic features of activity may persist in macroscopically inactive disease (14,15). Unfortunately, many clinicians continue to manage patients with UC symptomatically, thus the uptake of the T2T strategy in UC appears to be lagging (16).

The aim of this study is to provide a systematic review of the current research on T2T strategies in UC, discuss barriers to implementation, and offer practical advice on their incorporation into clinical care.

## METHODS

An electronic PubMed search from the time of the STRIDE systematic review up to March 31, 2018, was performed to assess accumulating evidence for the potential treatment targets of clinical factors, patient-reported outcomes (PROs), endoscopy, histology, imaging, and biomarkers (Table 1). Please see supplemental material for detailed methods (see Supplementary Digital Content 1, http://links.lww.com/AJG/A77).

**Table 1.**
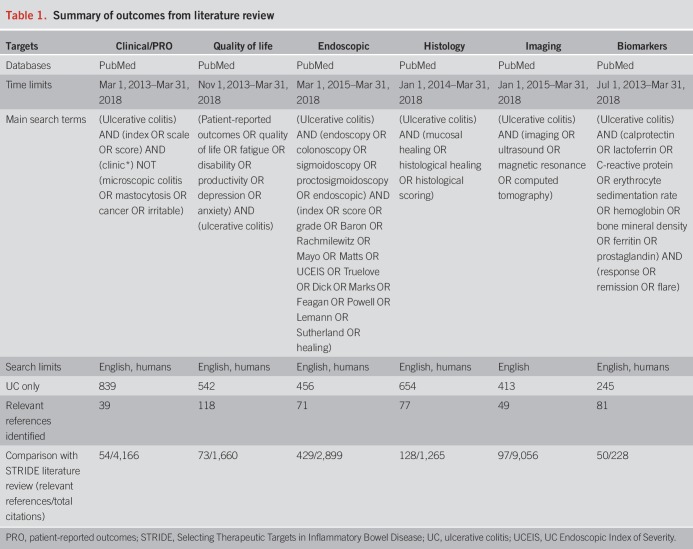
Summary of outcomes from literature review

The search strings largely replicated those used for the STRIDE publication (13). Selection criteria for inclusion of relevant publications were studies in patients with UC including placebo-controlled randomized clinical trials, interventional studies, observational studies, meta-analyses, and reviews. Case reports and studies performed in patients with cancer, neoplasia, and dysplasia were excluded.

## RESULTS

### Evolving targets since the publication of the STRIDE recommendations

**Figure 1.**
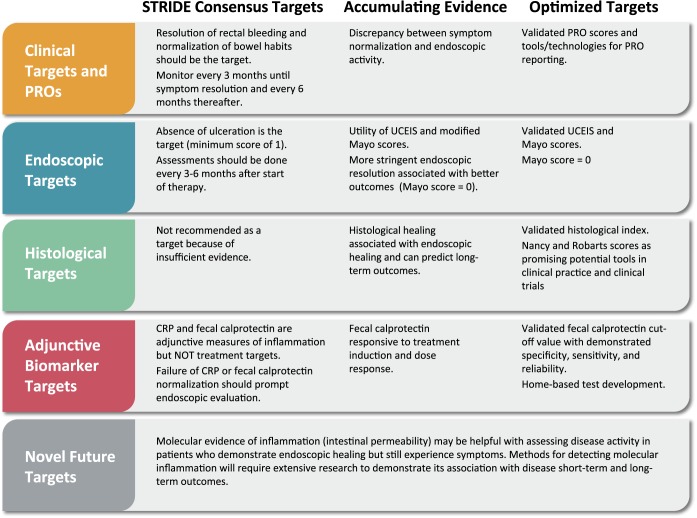
Accumulating evidence and evolution of specific targets in the management of UC. CRP, C-reactive protein; PRO, patient-reported outcomes; UCEIS, UC Endoscopic Index of Severity.

The STRIDE recommendations pointed to new IBD management standards, for UC in particular. This section highlights recent data that could help refine some of those recommendations to continue developing and improving UC treatments (Figure 1).

### Clinical targets and PROs

The STRIDE committee recommended that PROs, such as resolution of rectal bleeding and bowel habit normalization, should be a therapeutic target for UC. However, including objective inflammation measures as clinical study endpoints is important because the use of PROs alone has resulted in high remission rates for placebo (17). Furthermore, a small but consistent proportion of patients with endoscopic (Mayo score = 0) and histological remission may continue to report symptoms of unknown etiology (18–20). Noninflammatory mechanisms, such as small intestinal bacterial overgrowth, bile acid diarrhea, changes in motility or permeability, neurologic abnormalities, dysbiosis, or chronic fibrotic changes (21–23), may be possible causes. Conversely, around a quarter of patients who are clinically asymptomatic have endoscopically active disease (Mayo score > 1) (18). Interestingly, patients report a higher symptom burden than their healthcare providers using the same index; thus, the data collection method may be important to consider (24). Although simple surveys and/or mobile applications could improve symptom reporting by patients (24,25), these findings altogether point to the shortcomings of using solely clinical endpoints or PROs to reliably assess disease status. Given the US Food and Drug Administration's recognition of PROs as a clinical target, stool frequency and rectal bleeding remain important, although tools to monitor and quantify these measures need to be refined. Ultimately, evidence suggests that symptoms should be supplemented with objective targets.

### Quality of life measures

The restoration of a patient's quality of life (QoL) was considered to be the ultimate goal by the STRIDE committee (13). There are inherent challenges in using QoL endpoints, such as the lack of standardized instruments and the subjective nature of QoL. Two prospective studies using different instruments reported high-to-moderate correlation between QoL scores and clinical drug response over a short time (26,27). The disease-specific Inflammatory Bowel Disease Questionnaire measure was dose-responsive and had a linear correlation with Mayo scores (endoscopic score of disease activity). Therefore, although evidence of active disease association with reduced QoL continues to accumulate, consensus on QoL instruments across IBD studies remains a challenge.

Some clinicians have suggested that an objective disability index may be a valuable long-term target as well. In 2012, the IBD disability index was developed according to World Health Organization disability classifications (28). The instrument has since been validated, opening the door for measuring disability in clinical trials (29). In studies investigating factors associated with disability, active disease, poor drug adherence, and corticosteroid treatment (vs biological treatment) were associated with increased disability, supporting the utility of the IBD disability index (30,31).

Fatigue is commonly reported in patients with IBD and is associated with active disease; chronic fatigue has recently been shown to be more prevalent in patients with IBD than in a reference population (32–34). Fatigue has been associated with poor QoL using both general and IBD-specific instruments, emphasizing the importance of this PRO domain (32). However, validation of objective measures of fatigue is needed before incorporating it as a target in UC.

### Endoscopic targets

Endoscopic mucosal healing measurement is foundational to the indexes of disease severity and extent. The most often used endoscopic disease activity metrics are the UC Endoscopic Index of Severity (UCEIS) and the Mayo Clinic indexes (Mayo score) (13). Despite extensive research, these indexes are not fully validated and can be subject to interobserver disagreement (13,35–37). The UCEIS has shown less intra- and inter-reader variability than the Mayo score, but fewer studies of its predictive value and validity were available at the time of STRIDE (13). Since that time, studies have shown that UCEIS has a better correlation with disease severity and treatment responsiveness than the Mayo score and is more sensitive to detect deep ulcers becoming smaller and shallower, which the Mayo score overlooks (38,39). Recent Mayo score variations may surpass the original by incorporating the extent of inflammation along the colon while attempting to preserve the score's ease of use (40,41).

STRIDE preferred the Mayo score for real-world endoscopic healing evaluations in 2015, but emerging evidence supports UCEIS (13,38,39). For settings where the Mayo score is still preferred, centralization can improve interobserver agreement for the endoscopic components, and a recent study suggested that training can improve consistency in community settings (42,43).

When the STRIDE recommendations were developed, targets for the Mayo and UCEIS indexes were under debate, with a score of 1 considered the minimum target for both. Recent evidence suggests that more stringent endoscopic goals (i.e., Mayo or UCEIS score of 0) are associated with better outcomes and lower relapse risk (9.4% and 5.0%, respectively) (44–47).

Procedure type can also influence endoscopic assessments. Sigmoidoscopy is the standard technique in clinical trials, whereas colonoscopy is typically performed in clinical practice to confirm UC diagnosis and assess disease. A recent study demonstrated that sigmoidoscopy can evaluate distal colon inflammation with accuracy comparable with colonoscopy, particularly in patients with active disease (48).

Novel endoscopic imaging techniques (e.g., computed virtual chromoendoscopy, confocal laser endomicroscopy) may improve diagnostic accuracy for assessing endoscopic healing in UC (49). For example, confocal laser endomicroscopy can evaluate mucosal permeability that correlates with disease severity and treatment response (49–51). However, these imaging techniques require specialized training, and their utility in routine clinical care is still unclear.

### Histological targets

In UC, histological remission, defined as microscopic normalization of colonic mucosa, is distinct from endoscopic remission, which entails the resolution of endoscopically visible disease activity (13,14). Of several histology indexes available, the Nancy index and the Robarts Histopathology Index (RHI) have been the most studied indexes (52). In a prospective observational study, 87.1% of patients with histological remission at initial assessment remained in clinical remission after 1 year (53). In addition, histological remission in patients with UC was a strong predictor of steroid-free remission and clinical recurrence after 3 years of follow-up and was associated with lower hospitalization and corticosteroid use rates over a median follow-up period of 6 years (47,54,55). In a retrospective study, histological normalization was associated with increased odds of relapse-free survival compared with endoscopic healing or histological quiescence (56). In an observational cohort, the Geboes histology grade at baseline was strongly associated with a risk of clinical relapse in patients with UC who are in clinical remission after 12 months (57). Together, these data suggest that histologic remission can predict long-term outcomes.

Thus, including histological endpoints as treatment targets should enter into consideration (47). However, a uniform validated histology index is still needed because Ponte et al. (47) used the Nancy score, whereas the other studies used different indexes.

Validation of histologic indexes could broaden the use of this mucosal healing measure beyond its current limited application (13,58,59). The RHI was developed by selecting histopathological descriptors that had intra- and inter-reader reliability across the Geboes score, modified Riley score, and a visual analog scale. RHI incorporates the level of chronic inflammatory infiltrate, lamina propria neutrophils, neutrophils in the epithelium, and any erosion or ulceration present in the mucosal tissue (59). Similarly, the Nancy index scores ulceration, acute inflammatory cell infiltrate (i.e., neutrophils), and chronic inflammatory infiltrate (i.e., lymphocytes, plasmacytes) (58). These 2 indexes correlate with clinical remission and disease activity, as well as with the Mayo endoscopic score and fecal calprotectin (FC) concentrations (60). These indexes provide an opportunity for wider adoption of simplified or reliable histological scoring systems; however, further research is needed to validate their relationship with long-term outcomes, to establish clinically meaningful cutoff points, and to explore the feasibility and reliability of their practical adoption among community pathologists (52,58,59).

In the future, molecular studies may complement tissue exams for histological evaluation in UC. In this regard, intramucosal calprotectin was found to be associated with histological, endoscopic, and clinical remission (61).

### Imaging targets

Imaging modalities are an attractive monitoring alternative compared with the invasive current procedures but are not yet considered sufficient to evaluate mucosal healing in UC, novel methods notwithstanding (13). A magnetic resonance enterography disease index (magnetic resonance (MR) index of activity) was found to be viable to assess mucosal healing in a small cohort of patients with Crohn's disease (CD) (n = 48) (62). Laurent et al. (63) demonstrated that diffusion-weighted magnetic resonance imaging (MRI) using an MRI-specific index (Nancy score) accurately defined mucosal healing (endoscopically determined) in a small cohort of patients with UC (n = 29). Similarly, MR colonography was found to have a high accuracy for the diagnosis of disease activity and severity in UC (64). Further research to validate imaging modalities, indexes, and correlations with long-term disease outcomes are needed.

Ultrasound, a noninvasive radiation-free imaging modality used to evaluate the extent of disease activity (i.e., mucosal alterations, transmural involvement), was shown to have sensitivity and specificity similar to that of MRI and computed tomography for the diagnosis of IBD (65). Before the STRIDE publication, studies had investigated the ability of contrast-enhanced ultrasound to distinguish between quiescent and active disease *via* vascular activity (65). A systematic review on the utility of ultrasound for disease monitoring found that several UC ultrasound indexes have been developed, but they generally assessed bowel wall thickness, Doppler signal, wall layer stratification, compressibility, fatty wrapping, and strain pattern (66). The authors concluded that indexes have been developed with suboptimal methodology, thus development and validation of a new index are warranted (66).

### Biomarkers as targets

Although endoscopic and histological assessments are direct disease measures, they are invasive and costly, and thus noninvasive biomarkers of mucosal healing, treatment response, and/or disease flares are desirable (13). At the time of STRIDE, there was insufficient evidence supporting the use of biomarkers as surrogate endpoints for treatment optimization. However, data on the clinical utility of biomarkers, particularly FC, have been accumulating. Regular FC monitoring, with treatment escalation in patients with increased levels was associated with a reduced rate of relapse, albeit nonstatistically significant (67). In addition, mesalamine dose escalation reduced calprotectin levels to <100 μg/g, and relapse occurred sooner in patients with calprotectin level >200 μg/g (68).

New longitudinal observational studies found that escalating FC concentrations may predict relapse in patients with inactive UC as early as 3 months before the presentation of symptoms (69–75). Whether FC concentration changes can be used as surrogates of treatment response is still under investigation. Studies have reported that FC concentration reductions may be predictive of endoscopic and histological response to induction therapy and clinical remission (60,71,74,76–79). Low FC concentrations also correlate with the absence of mucosal inflammation or structural abnormalities (60,73,79). In addition, reductions in FC during treatment have been found to be dose-responsive (68,71,80,81). A meta-analysis by Mosli et al. (82) defined an optimum cutoff point for FC as 50 μg/g, but various concentration thresholds have been used across correlative studies. Thus, standardization and validation of a single FC cutoff point is needed to characterize its specificity and sensitivity as a biomarker ready for clinical practice. Because clinical data increasingly support FC as a UC biomarker, the optimal therapeutic target needs to be determined *via* well-designed disease-modification trials.

Studies are underway to identify and characterize additional promising fecal biomarkers such as leucine-rich α-2 glycoprotein, prostaglandin E-major urinary metabolite, hemoglobin concentration, M2-pyruvate kinase, lactoferrin, and high mobility group box 1 (81,83–86).

There are conflicting data on the utility of serological biomarkers as predictors of disease activity. C-reactive protein (CRP) and erythrocyte sedimentation rate were found to have low accuracy in detecting endoscopic activity in patients with UC (87). A *post hoc* analysis of a prospective clinical trial showed that CRP levels failed to discriminate between patients in clinical remission, with endoscopic inflammation and with mucosal healing (88). In pediatric patients with UC, neither marker was found to be useful in predicting clinical, endoscopic, or histological UC disease activity (89).

### Challenges and considerations for implementing T2T recommendations in clinical practice

**Figure 2.**
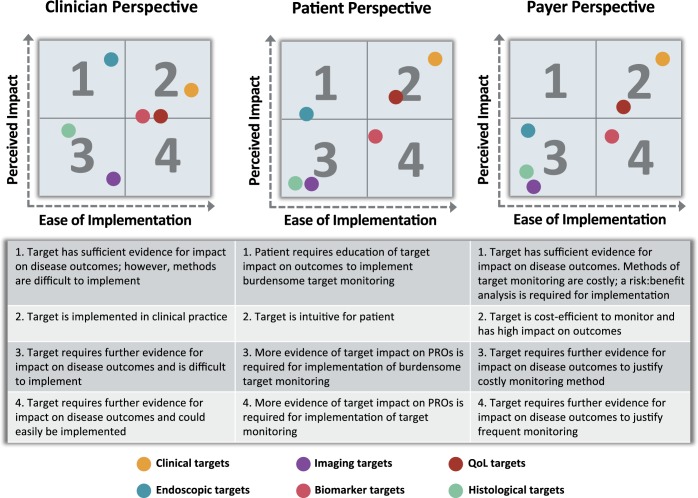
Feasibility of and barriers to implementation of a T2T approach. In the above matrices, the targets are placed on a scale that accounts for the ease of implementation as a treatment target (x-axis) and perceived impact of treatment target on disease outcome (y-axis). Quadrant 1 contains targets that are difficult to implement but have high perceived impact by stakeholder, quadrant 2 contains targets that are easy to implement and have high perceived impact, quadrant 3 contains targets that are difficult to implement and have low perceived impact, and finally quadrant 4 contains targets that are easy to implement and have low perceived impact. Each stakeholder will require different levels of evidence and education to successfully adopt the treat-to-target approach proposed by the STRIDE committee. Barriers to implementation are summarized under each matrix. PRO, patient-reported outcomes; QoL, quality of life; T2T, treat-to-target; STRIDE, Selecting Therapeutic Targets in Inflammatory Bowel Disease.

The T2T approaches in UC may require greater healthcare utilization, wider use of invasive procedures, and treatment escalation in the face of apparent symptomatic resolution, which raises potential barriers to implementation from patients, payers, and clinicians (Figure 2). Moreover, although the STRIDE consensus provided therapeutic goals, practical algorithms to reach these goals are needed. Thus, integration of T2T management into real-world UC clinical settings requires evidence generation to demonstrate its benefits and to validate therapeutic algorithms (2). In this section, we will highlight some of the implementation barriers and gaps in the evidence based on our review of the latest literature.

### Clinical perspective

Demonstrating that the T2T approach can modify the disease course and prevent disability and long-term complications is critical to justify the added costs and healthcare utilization (2). Even in CD, where the CALM trial demonstrated that a tight control algorithm could improve clinical and endoscopic outcomes, long-term follow-up was necessary to evaluate the impact on disease course and support a paradigm shift in management (90). Another study along similar lines is currently underway (Enhanced Algorithm for Crohn's Treatment Incorporating Early Combination Therapy; REACT2).

**Figure 3.**
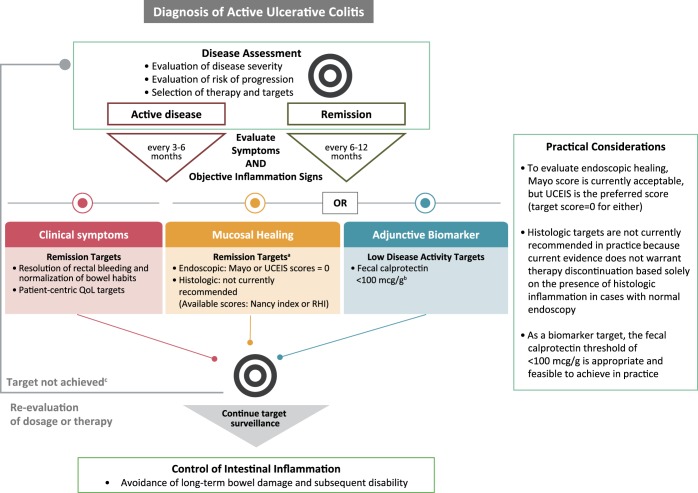
Proposed ulcerative colitis T2T algorithm. ^a^Mucosal healing as a treatment target must involve patient decision because of the high burden of monitoring and potential need for therapy escalation despite symptom resolution. ^b^Biomarker normalization as a treatment target must involve patient decision because of potential need for therapy escalation despite symptom resolution. ^c^If adjunctive biomarkers are not improving or normalizing, mucosal healing targets should be reassessed. QoL, quality of life; RHI, Robarts Histopathology Index; T2T, treat-to-target; UCEIS, UC Endoscopic Index of Severity.

For UC, based on the evidence summarized in this review, we propose an algorithm for incorporating T2T approaches into clinical care (Figure 3). However, this or any other algorithm would require prospective clinical studies to demonstrate its impact on disease outcomes and QoL.

Regarding the evaluation of endoscopic healing, the immaturity of the evidence connecting the pursuit of endoscopic targets to improved long-term outcomes represents a barrier for practical acceptance. This review captures the dichotomy between 2 disease score methods. As indicated in Figure 3, either Mayo or UCEIS can be used. In our opinion, UCEIS is the preferable score, although Mayo may be more familiar and therefore more feasible in clinical practice (for both, the target would be a score of 0). An important initial step to address current gaps, however, would be to aim for consistency in the routine adoption and recording of a disease score in patient reports, perhaps starting with Mayo, if that is the most feasible, but aspiring to eventually incorporate UCEIS as standard practice.

The incorporation of histologic scores lags behind endoscopic scores. Although it may be advisable to start considering how histologic evaluation could be integrated into routine practice, which we reflect in our algorithm in Figure 3, histologic score targets are not recommended for current practice because of the lack of prospective interventional studies demonstrating benefit of solely histologically guided therapy decisions. Given the limited number of current UC therapies, abandoning a medication in a patient with endoscopic remission and histologic inflammation only is not advisable until prospective data become available.

Because endoscopic scoring cannot be centralized in practice as it is in clinical trials, gaps in training represent another barrier to the effective adoption of endoscopic or histologic assessments. Educational initiatives or practice-centric programs guided by experts have proven useful in improving inter-reader reproducibility (42), but this is an area still in search of optimal solutions.

Given the invasiveness and cost of the monitoring procedures required, there is a need for data-driven evidence on the utility of noninvasive monitoring methods in predicting UC relapse to reduce healthcare and patient burden. At present, FC remains the most developed noninvasive means, and evidence suggests that it can be incorporated in the clinic for disease monitoring (91). A well-validated FC threshold that would indicate mucosal healing remains under investigation because clinical trials so far have used variable thresholds (13.9–261 μg/g) and correlative measures (e.g., reference data, definition of relapse) (71,81,88). Regarding practical application of FC testing, we propose that in current practice, a cutoff point of < 100 μg/g could be a target indicative of low disease activity (Figure 3). In practice, FC should be measured close to the time of an endoscopic assessment to “benchmark” the FC level to the individual patient. Furthermore, studies on home-based testing allowing patient self-measurement have reported good correlation with the classic enzyme-linked immunosorbent assay, which may help realize frequent FC monitoring with less patient burden (92). Imaging modalities offer a noninvasive method of monitoring disease activity for patients at higher risk for endoscopic disease and of tracking structural changes resulting from chronic inflammation that may be contributing to long-term complications. However, more research is required to investigate the specificity, sensitivity, and reliability of these tools.

Regarding PROs, 2 clear criteria have emerged as critically relevant for UC (rectal bleeding and stool frequency), but other QoL domains have been poorly studied (e.g., fatigue, disability) and are not consolidated into a single instrument. The increasing interest in PROs by regulators for drug development in IBD could and should propel the validation of tools following regulatory guidelines.

### Payer perspective

Payers will require clear and unequivocal assessments of the favorable risk-benefit ratio for T2T approaches vs the current status quo before this paradigm enters the mainstream of coverage. Ideally, these assessments would be supported by long-term randomized controlled trials, as well as adequate cost-effectiveness analysis. Evidence will be needed demonstrating that earlier aggressive treatment (or alternatively a more rapid step-up approach) and frequent monitoring may be more cost-effective in the long-term despite increased short-term costs (2). Obviously, the development of less costly and burdensome monitoring strategies would also lower the barrier to acceptance.

### Patient perspective

Patient considerations could be key to the success of personalized T2T approaches because motivated patients would be expected to remain adherent and compliant with protocols, even during times of disease remission and symptom resolution. Physicians should discuss specific goals that patients may have and patient concurrence with the treatment target. Patient adherence to a T2T approach will require their acceptance of dose escalation if the goal is deeper level healing or remission (histological or molecular/biomarker). Personalized regimens should consider disease severity and a patient's tolerance of aggressive treatment and possibly repeated procedures and testing, as well as the risk factors for complications, relapse, and side effects (2,93). Ideally, patient education would also foster the incorporation of lifestyle changes (dietary recommendations, etc.), which may have limited intrinsic efficacy but could contribute to symptom improvement (94).

Ultimately, the overarching aim of a T2T approach in UC is to meaningfully modify the disease course, restoring QoL and preventing major long-term functional impairment and disability. Therefore, measuring how T2T strategies deliver against specific goals under each perspective will be critical to validate this clinical paradigm and propel its wider adoption. Undoubtedly, such validation will require studies that are ambitious in scope (encompassing measures of clinical status, surgery rates, resource utilization, cost-effectiveness, patient function, QoL, and PROs), large in size, and lengthy in duration. Real-world cohorts may offer a good platform for such studies, although the challenges of conceptualizing comparative schemas (i.e., what would the reference controls for such a study be, and would historic data be valid) and reaching investigator consensus in the definition of suitable treatment targets and outcome measures should not be underestimated. Alternatively, large prospective clinical trials investigating the benefit of a T2T approach in UC, similar to that of CALM or REACT2 in CD, could help clinicians understand the value and feasibility of meeting targets with current therapies and monitoring tools.

### Future directions

As more is learned about intestinal inflammation, new tools and treatment targets may emerge. Endomicroscopy studies have developed more detailed mucosal healing criteria (including crypt numbers, crypt lumen deformity, crypt lumen leakage, and vascular leakage) (95). Further studies could determine the predictive value of endomicroscopic mucosal changes regarding clinical outcomes. The search for biomarkers is also evolving, with a recent study identifying 4 gene transcripts responsive to antitumor necrosis factor therapy and correlated with endoscopic disease activity; these molecular markers pinpoint changes in disease activity more accurately than CRP, erythrocyte sedimentation rate, and platelet count (96). Further research is needed to shed light on the underlying causes and etiology of persistent symptoms in patients with endoscopic remission (20,23). To that end, the development of a functional UC bowel damage index beyond endoscopy or histology scoring (akin to the Lémann score in CD (97)) would provide a major research and management tool.

The T2T paradigm, widely accepted in rheumatoid arthritis, is an emerging approach in IBD. This approach is currently more established in the treatment of CD, but growing evidence supports its usefulness in UC. Given the new evidence, the T2T recommendations (STRIDE) could be updated for both CD and UC (98). In the near future, we might need to look beyond the mucosa and recognize fibrosis and molecular healing as components of UC. All these factors may hold the key to avoiding long-term functional deficits and disability in UC.

Finally, we would be remiss to deny that the implementation of T2T strategies in routine practice remains challenging and requires a shift. Successful T2T implementation will require patient and physician education and communication (to create true personalized treatment plans and goals), renewed efforts in evidence generation to validate reliable and preferably noninvasive endpoints that predict favorable long-term outcomes, and establishment of the superior risk-benefit and cost-effectiveness profile of a T2T strategy over the current paradigms.

## CONFLICTS OF INTEREST

**Guarantor of the article:** Ryan Ungaro, MD, and Jean-Frédéric Colombel, MD.

**Specific author contributions:** R.U. and L.P.-B.: screening of literature search output and study selection. All authors contributed to the conception and design, contributed to the critical review and revision of each draft of the manuscript, and approved the final version for submission.

**Financial support:** Research support from Boehringer Ingelheim, Abbvie, and Pfizer. Supported by a Career Development Award from the Crohn's and Colitis Foundation. R.U. has served as a consultant and/or advisory board member for Takeda, Pfizer, and Janssen. J.-F.C. has received grant support from AbbVie, Janssen Pharmaceuticals, and Takeda; has served as a speaker for AbbVie, Amgen, and Ferring Pharmaceuticals; has served as a consultant for AbbVie, Amgen, Boehringer Ingelheim, Celgene Corporation, Celltrion, Eli Lilly, Enterome, Ferring Pharmaceuticals, Genentech, Janssen Pharmaceuticals, MedImmune, Merck & Co., Pfizer, Protagonist, Second Genome, Seres Therapeutics, Shire, Takeda, and Theradiag; and is a shareholder of Genfit and Intestinal Biotech Development. T.L. is an employee of Takeda Pharmaceuticals U.S.A., Inc. L.P.-B. has received consulting fees from Merck, Abbott, Janssen, Genentech, Mitsubishi, Ferring, Norgine, Tillotts, Vifor, Shire, Therakos, Pharmacosmos, Pilège, BMS, UCB Pharma, Hospira, Celltrion, Takeda, Boehringer Ingelheim, Lilly, and Pfizer; and has received lecture fees from Merck, Abbott, Janssen, Ferring, Norgine, Tillotts, Vifor, Therakos, and HAC Pharma.

**Potential competing interests:** R.U. has served as a consultant and/or Advisory Board member for Takeda, Pfizer, and Janssen. J.-F.C. has received grant support from AbbVie, Janssen Pharmaceuticals, and Takeda; has served as a speaker for AbbVie, Amgen, and Ferring Pharmaceuticals; has served as a consultant for AbbVie, Amgen, Boehringer Ingelheim, Celgene Corporation, Celltrion, Eli Lilly, Enterome, Ferring Pharmaceuticals, Genentech, Janssen Pharmaceuticals, MedImmune, Merck & Co., Pfizer, Protagonist, Second Genome, Seres Therapeutics, Shire, Takeda, and Theradiag; and is a shareholder of Genfit and Intestinal Biotech Development. T.L. is an employee of Takeda Pharmaceuticals U.S.A., Inc. L.P.-B. has received consulting fees from Merck, Abbott, Janssen, Genentech, Mitsubishi, Ferring, Norgine, Tillotts, Vifor, Shire, Therakos, Pharmacosmos, Pilège, BMS, UCB Pharma, Hospira, Celltrion, Takeda, Boehringer Ingelheim, Lilly, and Pfizer; and has received lecture fees from Merck, Abbott, Janssen, Ferring, Norgine, Tillotts, Vifor, Therakos, and HAC Pharma. Medical writing and editing assistance for this article was provided by Reem Berro, PhD, Jessica Cardenas, PhD, and Katy Stevens, PhD, of inVentiv Medical Communications, LLC, and a Syneos Health Group company, respectively, and was supported by Takeda Pharmaceuticals U.S.A., Inc.

## References

[1] Ungaro R Mehandru S Allen PB Ulcerative colitis. Lancet 2017;389:1756–70. 27914657 10.1016/S0140-6736(16)32126-2 PMC6487890

[2] Colombel JF Narula N Peyrin-Biroulet L Management strategies to improve outcomes of patients with inflammatory bowel diseases. Gastroenterology 2017;152:351–61.e5. 27720840 10.1053/j.gastro.2016.09.046

[3] Dotti I Mora-Buch R Ferrer-Picon E Alterations in the epithelial stem cell compartment could contribute to permanent changes in the mucosa of patients with ulcerative colitis. Gut 2017;66:2069–79. 27803115 10.1136/gutjnl-2016-312609 PMC5749340

[4] Ordás I Eckmann L Talamini M Ulcerative colitis. Lancet 2012;380(9853):1606–19. 22914296 10.1016/S0140-6736(12)60150-0

[5] Torres J Billioud V Sachar DB Ulcerative colitis as a progressive disease: The forgotten evidence. Inflamm Bowel Dis 2012;18:1356–63. 22162423 10.1002/ibd.22839

[6] Roda G Narula N Pinotti R Systematic review with meta-analysis: Proximal disease extension in limited ulcerative colitis. Aliment Pharmacol Ther 2017;45:1481–92. 28449361 10.1111/apt.14063 PMC6350510

[7] Fumery M Singh S Dulai PS Natural history of adult ulcerative colitis in population-based cohorts: A systematic review. Clin Gastroenterol Hepatol 2018;16:343–56.e3. 28625817 10.1016/j.cgh.2017.06.016 PMC6658168

[8] Eriksson C Cao Y Rundquist S Changes in medical management and colectomy rates: A population-based cohort study on the epidemiology and natural history of ulcerative colitis in Orebro, Sweden, 1963-2010. Aliment Pharmacol Ther 2017;46:748–57. 28833287 10.1111/apt.14268

[9] Darr U Khan N Treat to target in inflammatory bowel disease: An updated review of literature. Curr Treat Options Gastroenterol 2017;15:116–25. 28161818 10.1007/s11938-017-0130-6

[10] Peyrin-Biroulet L Germain A Patel AS Systematic review: Outcomes and post-operative complications following colectomy for ulcerative colitis. Aliment Pharmacol Ther 2016;44:807–16. 27534519 10.1111/apt.13763

[11] Pineton de Chambrun G Peyrin-Biroulet L Lémann M Clinical implications of mucosal healing for the management of IBD. Nat Rev Gastroenterol Hepatol 2010;7:15–29. 19949430 10.1038/nrgastro.2009.203

[12] Dassopoulos T Cohen RD Scherl EJ Ulcerative colitis care pathway. Gastroenterology 2015;149:238–45. 26025078 10.1053/j.gastro.2015.05.036

[13] Peyrin-Biroulet L Sandborn W Sands BE Selecting therapeutic targets in inflammatory bowel disease (STRIDE): Determining therapeutic goals for treat-to-target. Am J Gastroenterol 2015;110:1324–38. 26303131 10.1038/ajg.2015.233

[14] Peyrin-Biroulet L Bressenot A Kampman W Histologic remission: The ultimate therapeutic goal in ulcerative colitis? Clin Gastroenterol Hepatol 2014;12:929–34.e2. 23911875 10.1016/j.cgh.2013.07.022

[15] Arijs I De Hertogh G Lemmens B Effect of vedolizumab (anti-alpha4beta7-integrin) therapy on histological healing and mucosal gene expression in patients with UC. Gut 2018;67:43–52. 27802155 10.1136/gutjnl-2016-312293

[16] Bryant RV Costello SP Schoeman S Limited uptake of ulcerative colitis “treat to target” recommendations in real-world practice. J Gastroenterol Hepatol 2018;33:599–607. 28806471 10.1111/jgh.13923

[17] Jairath V Khanna R Zou GY Development of interim patient-reported outcome measures for the assessment of ulcerative colitis disease activity in clinical trials. Aliment Pharmacol Ther 2015;42:1200–10. 26388424 10.1111/apt.13408

[18] Colombel JF Keir ME Scherl A Discrepancies between patient-reported outcomes, and endoscopic and histological appearance in UC. Gut 2017;66:2063–8. 27590995 10.1136/gutjnl-2016-312307 PMC5749342

[19] Jharap B Sandborn WJ Reinisch W Randomised clinical study: Discrepancies between patient-reported outcomes and endoscopic appearance in moderate to severe ulcerative colitis. Aliment Pharmacol Ther 2015;42:1082–92. 26381802 10.1111/apt.13387 PMC5049645

[20] Teruel C Garrido E Mesonero F Diagnosis and management of functional symptoms in inflammatory bowel disease in remission. World J Gastrointest Pharmacol Ther 2016;7:78–90. 26855814 10.4292/wjgpt.v7.i1.78 PMC4734957

[21] Brochard C Siproudhis L Ropert A Anorectal dysfunction in patients with ulcerative colitis: Impaired adaptation or enhanced perception? Neurogastroenterol Motil 2015;27:1032–7. 25940976 10.1111/nmo.12580

[22] Loening-Baucke V Metcalf AM Shirazi S Anorectal manometry in active and quiescent ulcerative colitis. Am J Gastroenterol 1989;84:892–7. 2756980

[23] Colombel JF Shin A Gibson PR AGA Clinical Practice Update on functional gastrointestinal symptoms in patients with inflammatory bowel disease. Clin Gastroenterol Hepatol 2019;17:380–90.e1. 30099108 10.1016/j.cgh.2018.08.001 PMC6581193

[24] Bennebroek Evertsz' F Nieuwkerk PT Stokkers PC The patient simple clinical colitis activity index (P-SCCAI) can detect ulcerative colitis (UC) disease activity in remission: A comparison of the P-SCCAI with clinician-based SCCAI and biological markers. J Crohns Colitis 2013;7:890–900. 23269224 10.1016/j.crohns.2012.11.007

[25] Van Deen WK van der Meulen-de Jong AE Parekh NK Development and validation of an inflammatory bowel diseases monitoring index for use with mobile health technologies. Clin Gastroenterol Hepatol 2016;14:1742–50.e7. 26598228 10.1016/j.cgh.2015.10.035

[26] Panés J Su C Bushmakin AG Randomized trial of tofacitinib in active ulcerative colitis: Analysis of efficacy based on patient-reported outcomes. BMC Gastroenterol 2015;15:14. 25651782 10.1186/s12876-015-0239-9 PMC4323227

[27] Yarlas AYL Hodgkins P The relationship among multiple patient-reported outcomes measures for patients with ulcerative colitis receiving treatment with MMX ® formulated delayed-release mesalamine. Qual Life Res 2015;24:671–83. 25193617 10.1007/s11136-014-0797-2 PMC4349951

[28] Peyrin-Biroulet L Cieza A Sandborn WJ Development of the first disability index for inflammatory bowel disease based on the international classification of functioning, disability and health. Gut 2012;61:241–7. 21646246 10.1136/gutjnl-2011-300049 PMC3245899

[29] Gower-Rousseau C Sarter H Savoye G Validation of the inflammatory bowel disease disability index in a population-based cohort. Gut 2017;66:588–96. 26646934 10.1136/gutjnl-2015-310151

[30] Lo B Prosberg MV Gluud LL Systematic review and meta-analysis: Assessment of factors affecting disability in inflammatory bowel disease and the reliability of the inflammatory bowel disease disability index. Aliment Pharmacol Ther 2018;47:6–15. 28994131 10.1111/apt.14373

[31] Yoon JY Shin JE Park SH Disability due to inflammatory bowel disease is correlated with drug compliance, disease activity, and quality of life. Gut Liver 2017;11:370–6. 28208008 10.5009/gnl16422 PMC5417779

[32] Cohen BL Zoëga H Shah SA Fatigue is highly associated with poor health-related quality of life, disability and depression in newly-diagnosed patients with inflammatory bowel disease, independent of disease activity. Aliment Pharmacol Ther 2014;39:811–22. 24612278 10.1111/apt.12659 PMC4670472

[33] Opheim R Fagermoen MS Bernklev T Fatigue interference with daily living among patients with inflammatory bowel disease. Qual Life Res 2014;23:707–17. 23975381 10.1007/s11136-013-0508-4

[34] Huppertz-Hauss G Hoivik ML Jelsness-Jorgensen LP Fatigue in a population-based cohort of patients with inflammatory bowel disease 20 years after diagnosis: The IBSEN study. Scand J Gastroenterol 2017;52:351–8. 27852169 10.1080/00365521.2016.1256425

[35] Walsh AJ Ghosh A Brain AO Comparing disease activity indices in ulcerative colitis. J Crohns Colitis 2014;8:318–25. 24120021 10.1016/j.crohns.2013.09.010

[36] Mohammed Vashist N Samaan M Mosli MH Endoscopic scoring indices for evaluation of disease activity in ulcerative colitis. Cochrane Database Syst Rev 2018;1:CD011450. 29338066 10.1002/14651858.CD011450.pub2 PMC6491285

[37] Tontini GE Bisschops R Neumann H Endoscopic scoring systems for inflammatory bowel disease: Pros and cons. Expert Rev Gastroenterol Hepatol 2014;8:543–54. 24650249 10.1586/17474124.2014.899899

[38] Arai M Naganuma M Sugimoto S The Ulcerative Colitis Endoscopic Index of Severity is useful to predict medium- to long-term prognosis in ulcerative colitis patients with clinical remission. J Crohns Colitis 2016;10:1303–9. 27194529 10.1093/ecco-jcc/jjw104

[39] Ikeya K Hanai H Sugimoto K The ulcerative colitis endoscopic index of severity more accurately reflects clinical outcomes and long-term prognosis than the Mayo endoscopic score. J Crohns Colitis 2016;10:286–95. 26581895 10.1093/ecco-jcc/jjv210 PMC4957474

[40] Lobatón T Bessissow T De Hertogh G The modified Mayo endoscopic score (MMES): A new index for the assessment of extension and severity of endoscopic activity in ulcerative colitis patients. J Crohns Colitis 2015;9:846–52. 26116558 10.1093/ecco-jcc/jjv111

[41] Balint A Farkas K Szepes Z How disease extent can be included in the endoscopic activity index of ulcerative colitis: The panMayo score, a promising scoring system. BMC Gastroenterol 2018;18:7. 29310593 10.1186/s12876-017-0725-3 PMC5759871

[42] Daperno M Comberlato M Bossa F Training programs on endoscopic scoring systems for inflammatory bowel disease lead to a significant increase in interobserver agreement among community gastroenterologists. J Crohns Colitis 2017;11:556–61. 28453758 10.1093/ecco-jcc/jjw181

[43] Feagan BG Sandborn WJ D'Haens G The role of centralized reading of endoscopy in a randomized controlled trial of mesalamine for ulcerative colitis. Gastroenterology 2013;145:149–57 e2. 23528626 10.1053/j.gastro.2013.03.025

[44] Barreiro-de Acosta M Vallejo N de la Iglesia D Evaluation of the risk of relapse in ulcerative colitis according to the degree of mucosal healing (Mayo 0 vs 1): A longitudinal cohort study. J Crohns Colitis 2016;10:13–9. 26351390 10.1093/ecco-jcc/jjv158

[45] Boal Carvalho P Dias de Castro F Rosa B Mucosal healing in ulcerative colitis: When zero is better. J Crohns Colitis 2016;10:20–5. 26438714 10.1093/ecco-jcc/jjv180

[46] Kim JH Cheon JH Park Y Effect of mucosal healing (Mayo 0) on clinical relapse in patients with ulcerative colitis in clinical remission. Scand J Gastroenterol 2016;51:1069–74. 26895215 10.3109/00365521.2016.1150503

[47] Ponte A Pinho R Fernandes S Impact of histological and endoscopic remissions on clinical recurrence and recurrence-free time in ulcerative colitis. Inflamm Bowel Dis 2017;23:2238–44. 28991857 10.1097/MIB.0000000000001275

[48] Colombel JF Ordas I Ullman T Agreement between rectosigmoidoscopy and colonoscopy analyses of disease activity and healing in patients with ulcerative colitis. Gastroenterology 2016;150:389–95.e3. 26526713 10.1053/j.gastro.2015.10.016

[49] Karstensen JG Saftoiu A Brynskov J Confocal laser endomicroscopy in ulcerative colitis: A longitudinal study of endomicroscopic changes and response to medical therapy (with videos). Gastrointest Endosc 2016;84:279–86.e1. 26945556 10.1016/j.gie.2016.01.069

[50] Karstensen JG Evaluation of confocal laser endomicroscopy for assessment and monitoring of therapeutic response in patients with inflammatory bowel disease. Dan Med J 2016;63:B5301. 27808042

[51] Chang J Leong RW Wasinger VC Impaired intestinal permeability contributes to ongoing bowel symptoms in patients with inflammatory bowel disease and mucosal healing. Gastroenterology 2017;153:723–31.e1. 28601482 10.1053/j.gastro.2017.05.056

[52] Mosli MH Parker CE Nelson SA Histologic scoring indices for evaluation of disease activity in ulcerative colitis. Cochrane Database Syst Rev 2017;5:CD011256. 28542712 10.1002/14651858.CD011256.pub2 PMC6481362

[53] Narang V Kaur R Garg B Association of endoscopic and histological remission with clinical course in patients of ulcerative colitis. Intest Res 2018;16:55–61. 29422798 10.5217/ir.2018.16.1.55 PMC5797272

[54] Frieri G Galletti B Di Ruscio M The prognostic value of histology in ulcerative colitis in clinical remission with mesalazine. Therap Adv Gastroenterol 2017;10:749–59. 10.1177/1756283X17722926 PMC5638180 29051786

[55] Bryant RV Burger DC Delo J Beyond endoscopic mucosal healing in UC: Histological remission better predicts corticosteroid use and hospitalisation over 6 years of follow-up. Gut 2016;65:408–14. 25986946 10.1136/gutjnl-2015-309598

[56] Christensen B Hanauer SB Erlich J Histologic normalization occurs in ulcerative colitis and is associated with improved clinical outcomes. Clin Gastroenterol Hepatol 2017;15:1557–64.e1. 28238954 10.1016/j.cgh.2017.02.016 PMC5618439

[57] Zenlea T Yee EU Rosenberg L Histology grade is independently associated with relapse risk in patients with ulcerative colitis in clinical remission: A prospective study. Am J Gastroenterol 2016;111:685–90. 26977756 10.1038/ajg.2016.50

[58] Marchal-Bressenot A Salleron J Boulagnon-Rombi C Development and validation of the Nancy histological index for UC. Gut 2017;66:43–9. 26464414 10.1136/gutjnl-2015-310187

[59] Mosli MH Feagan BG Zou G Development and validation of a histological index for UC. Gut 2017;66:50–8. 26475633 10.1136/gutjnl-2015-310393

[60] Magro F Lopes J Borralho P Comparison of different histological indexes in the assessment of UC activity and their accuracy regarding endoscopic outcomes and faecal calprotectin levels. Gut 2018 [Epub ahead of print February 3, 2018.] 10.1136/gutjnl-2017-315545 29437913

[61] Guirgis M Wendt E Wang LM Beyond histological remission: Intramucosal calprotectin as a potential predictor of outcomes in ulcerative colitis. J Crohns Colitis 2017;11:460–7. 27856523 10.1093/ecco-jcc/jjw174

[62] Ordas I Rimola J Rodriguez S Accuracy of magnetic resonance enterography in assessing response to therapy and mucosal healing in patients with Crohn's disease. Gastroenterology 2014;146:374–82.e1. 24177375 10.1053/j.gastro.2013.10.055

[63] Laurent V Naude S Vuitton L Accuracy of diffusion-weighted magnetic resonance colonography in assessing mucosal healing and the treatment response in patients with ulcerative colitis. J Crohns Colitis 2017;11:716–23. 27932450 10.1093/ecco-jcc/jjw211

[64] Ordás I Rimola J García-Bosch O Diagnostic accuracy of magnetic resonance colonography for the evaluation of disease activity and severity in ulcerative colitis: A prospective study. Gut 2013;62:1566–72. 22936673 10.1136/gutjnl-2012-303240

[65] Knieling F Waldner MJ Light and sound—emerging imaging techniques for inflammatory bowel disease. World J Gastroenterol 2016;22:5642–54. 27433080 10.3748/wjg.v22.i25.5642 PMC4932202

[66] Bots S Nylund K Lowenberg M Ultrasound for assessing disease activity in IBD patients: A systematic review of activity scores. J Crohns Colitis 2018;12:920–9. 29684200 10.1093/ecco-jcc/jjy048

[67] Lasson A Öhman L Stotzer PO Pharmacological intervention based on fecal calprotectin levels in patients with ulcerative colitis at high risk of a relapse: A prospective, randomized, controlled study. United Eur Gastroenterol J 2015;3:72–9. 10.1177/2050640614560785 PMC4315686 25653861

[68] Osterman MT Aberra FN Cross R Mesalamine dose escalation reduces fecal calprotectin in patients with quiescent ulcerative colitis. Clin Gastroenterol Hepatol 2014;12:1887–93.e3. 24793028 10.1016/j.cgh.2014.03.035 PMC4214893

[69] Burri E Beglinger C von Felten S Fecal calprotectin and the clinical activity index are both useful to monitor medical treatment in patients with ulcerative colitis. Dig Dis Sci 2015;60:485–91. 25344905 10.1007/s10620-014-3383-0

[70] De Vos M Louis EJ Jahnsen J Consecutive fecal calprotectin measurements to predict relapse in patients with ulcerative colitis receiving infliximab maintenance therapy. Inflamm Bowel Dis 2013;19:2111–7. 23883959 10.1097/MIB.0b013e31829b2a37

[71] Kostas A Siakavellas SI Kosmidis C Fecal calprotectin measurement is a marker of short-term clinical outcome and presence of mucosal healing in patients with inflammatory bowel disease. World J Gastroenterol 2017;23:7387–96. 29151692 10.3748/wjg.v23.i41.7387 PMC5685844

[72] Molander P Farkkila M Ristimaki A Does fecal calprotectin predict short-term relapse after stopping TNFalpha-blocking agents in inflammatory bowel disease patients in deep remission?. J Crohns Colitis 2015;9:33–40. 25052347 10.1016/j.crohns.2014.06.012

[73] Theede K Holck S Ibsen P Fecal calprotectin predicts relapse and histological mucosal healing in ulcerative colitis. Inflamm Bowel Dis 2016;22:1042–8. 26919460 10.1097/MIB.0000000000000736

[74] Yamamoto T Shimoyama T Matsumoto K Consecutive monitoring of faecal calprotectin during mesalazine suppository therapy for active rectal inflammation in ulcerative colitis. Aliment Pharmacol Ther 2015;42:549–58. 26140337 10.1111/apt.13308

[75] Zhulina Y Cao Y Amcoff K The prognostic significance of faecal calprotectin in patients with inactive inflammatory bowel disease. Aliment Pharmacol Ther 2016;44:495–504. 27402063 10.1111/apt.13731

[76] Sandborn WJ Panes J Zhang H Correlation between concentrations of fecal calprotectin and outcomes of patients with ulcerative colitis in a phase 2 trial. Gastroenterology 2016;150:96–102. 26376350 10.1053/j.gastro.2015.09.001

[77] Kristensen V Røseth A Ahmad T Fecal calprotectin: A reliable predictor of mucosal healing after treatment for active ulcerative colitis. Gastroenterol Res Pract 2017;2017:2098293. 29225617 10.1155/2017/2098293 PMC5684574

[78] Patel A Panchal H Dubinsky MC Fecal calprotectin levels predict histological healing in ulcerative colitis. Inflamm Bowel Dis 2017;23:1600–4. 28590341 10.1097/MIB.0000000000001157

[79] Zittan E Kelly OB Kirsch R Low fecal calprotectin correlates with histological remission and mucosal healing in ulcerative colitis and colonic Crohn's disease. Inflamm Bowel Dis 2016;22:623–30. 26829408 10.1097/MIB.0000000000000652

[80] D'Haens G Ferrante M Vermeire S Fecal calprotectin is a surrogate marker for endoscopic lesions in inflammatory bowel disease. Inflamm Bowel Dis 2012;18:2218–24. 22344983 10.1002/ibd.22917

[81] Frin AC Filippi J Boschetti G Accuracies of fecal calprotectin, lactoferrin, M2-pyruvate kinase, neopterin and zonulin to predict the response to infliximab in ulcerative colitis. Dig Liver Dis 2017;49:11–6. 27693318 10.1016/j.dld.2016.09.001

[82] Mosli MH Zou G Garg SK C-reactive protein, fecal calprotectin, and stool lactoferrin for detection of endoscopic activity in symptomatic inflammatory bowel disease patients: A systematic review and meta-analysis. Am J Gastroenterol 2015;110:802–19. 25964225 10.1038/ajg.2015.120

[83] Arai Y Matsuura T Matsuura M Prostaglandin E-major urinary metabolite as a biomarker for inflammation in ulcerative colitis: Prostaglandins revisited. Digestion 2016;93:32–9. 26788915 10.1159/000441665

[84] Hiraoka S Kato J Nakarai A Consecutive measurements by faecal immunochemical test in quiescent ulcerative colitis patients can detect clinical relapse. J Crohns Colitis 2016;10:687–94. 26802083 10.1093/ecco-jcc/jjw025

[85] Palone F Vitali R Cucchiara S Fecal HMGB1 reveals microscopic inflammation in adult and pediatric patients with inflammatory bowel disease in clinical and endoscopic remission. Inflamm Bowel Dis 2016;22:2886–93. 27755215 10.1097/MIB.0000000000000938

[86] Shinzaki S Matsuoka K Iijima H Leucine-rich alpha-2 glycoprotein is a serum biomarker of mucosal healing in ulcerative colitis. J Crohns Colitis 2017;11:84–91. 27466171 10.1093/ecco-jcc/jjw132 PMC5175492

[87] Miranda-Garcia P Chaparro M Gisbert JP Correlation between serological biomarkers and endoscopic activity in patients with inflammatory bowel disease. Gastroenterol Hepatol 2016;39:508–15. 27020243 10.1016/j.gastrohep.2016.01.015

[88] Langhorst J Boone J Lauche R Faecal lactoferrin, calprotectin, PMN-elastase, CRP, and white blood cell count as indicators for mucosal healing and clinical course of disease in patients with mild to moderate ulcerative colitis: Post hoc analysis of a prospective clinical trial. J Crohns Colitis 2016;10:786–94. 26874351 10.1093/ecco-jcc/jjw044

[89] Alper A Zhang L Pashankar DS Correlation of erythrocyte sedimentation rate and C-reactive protein with pediatric inflammatory bowel disease activity. J Pediatr Gastroenterol Nutr 2017;65:e25–e27. 27741061 10.1097/MPG.0000000000001444

[90] Colombel JF Panaccione R Bossuyt P Effect of tight control management on Crohn's disease (CALM): A multicentre, randomised, controlled phase 3 trial. Lancet 2018;390:2779–89. 29096949 10.1016/S0140-6736(17)32641-7

[91] Abej E El-Matary W Singh H The utility of fecal calprotectin in the real-world clinical care of patients with inflammatory bowel disease. Can J Gastroenterol Hepatol 2016;2016:2483261. 27774443 10.1155/2016/2483261 PMC5059522

[92] Bello C Roseth A Guardiola J Usability of a home-based test for the measurement of fecal calprotectin in asymptomatic IBD patients. Dig Liver Dis 2017;49:991–6. 28587751 10.1016/j.dld.2017.05.009

[93] Fries W Viola A Manetti N Disease patterns in late-onset ulcerative colitis: Results from the IG-IBD AGED study. Dig Liver Dis 2017;49:17–23. 27717794 10.1016/j.dld.2016.09.006

[94] Nikolaus S Schreiber S Siegmund B Patient education in a 14-month randomised trial fails to improve adherence in ulcerative colitis: Influence of demographic and clinical parameters on non-adherence. J Crohns Colitis 2017;11:1052–62. 28486634 10.1093/ecco-jcc/jjx062

[95] Hundorfean G Chiriac MT Mihai S Development and validation of a confocal laser endomicroscopy-based score for in vivo assessment of mucosal healing in ulcerative colitis patients. Inflamm Bowel Dis 2017;24:35–44. 29272480 10.1093/ibd/izx012

[96] Planell N Masamunt MC Leal RF Usefulness of transcriptional blood biomarkers as a non-invasive surrogate marker of mucosal healing and endoscopic response in ulcerative colitis. J Crohns Colitis 2017;11:1335–46. 28981629 10.1093/ecco-jcc/jjx091 PMC5881703

[97] Pariente B Cosnes J Danese S Development of the Crohn's disease digestive damage score, the Lémann score. Inflamm Bowel Dis 2011;17:1415–22. 21560202 10.1002/ibd.21506 PMC3116198

[98] Pouillon L Peyrin-Biroulet L It is time to revise the STRIDE guidelines determining therapeutic goals for treat-to-target in inflammatory bowel disease. J Crohns Colitis 2018;12:509. 29309565 10.1093/ecco-jcc/jjx174

